# Approaching the Intrinsic Limits of Short Channel Vertical Organic Electrochemical Transistors

**DOI:** 10.1021/acsami.4c02772

**Published:** 2024-08-20

**Authors:** Alvaro Galeana Perez Negron, Andreas Schander, Michael Skowrons, Henrique Frulani de Paula Barbosa, Björn Lüssem

**Affiliations:** † Institut für Mikrosensoren, -Aktoren, und -Systeme (IMSAS), Universität Bremen, 28359 Bremen, Germany; ‡ Department of Physics, Kent State University, Kent, Ohio 44240, United States

**Keywords:** organic electrochemical transistors, parasitic resistance, vertical architecture, four-point structures, intrinsic transconductance, electrodeposition

## Abstract

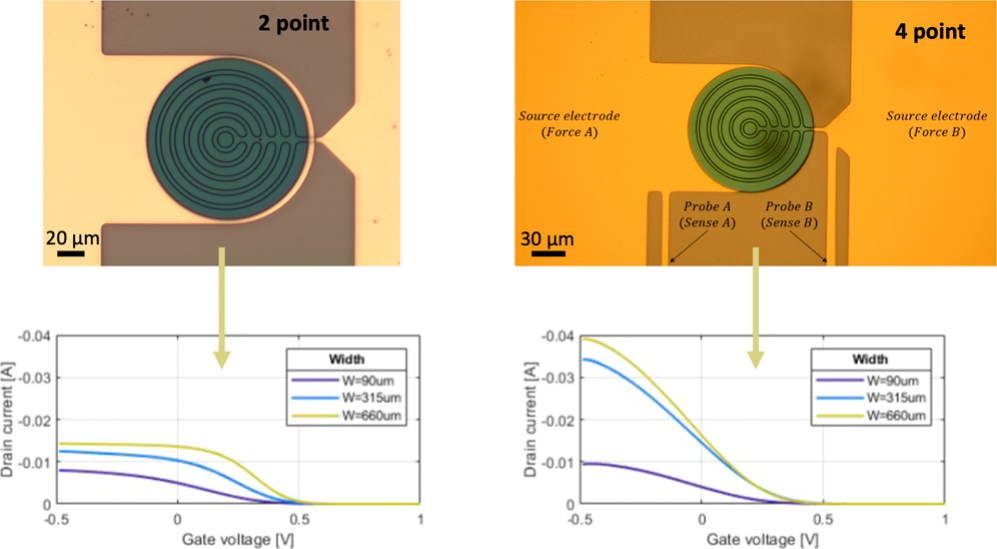

Vertical architectures for organic electrochemical transistors (OECTs), due to their submicrometer channel lengths, have presented themselves as a straightforward design approach for achieving high *g*_m_/τ ratios, a figure of merit that assesses the performance of the devices by virtue of their transconductance (*g*_m_ = d*I*_D_/d*V*_GS_) and switching time constant (τ). However, as the practical limitations of the geometries are overcome, the influence of parasitic phenomena becomes more dominant and limits the performance of the device. One approach to reduce the detrimental effects of parasitic resistance in the drain-source circuit is to use a four-point sourcing technique. Here, vertical OECTs are fabricated with four-point structures to approach the intrinsic limit of these devices. It is shown that this approach improves the saturation behavior of the devices, closing the gap between measured *g*_m_ and intrinsic transconductance *g*_mi_ at their peak values. Overall, the results discussed here provide insight into the effects of parasitic resistance on OECTs, which in contrast to field-effect transistors, are not as extensively documented.

## 1 Introduction

Organic electrochemical transistors (OECTs) are biocompatible transducers that have emerged as a promising biosensing technology^1^ due to their ability to detect and amplify signals in aqueous environments.^2^ They operate by coupling an electronic circuit with an ionic circuit through an organic mixed-ion-electron semiconductor as a channel material.

The mixed conduction inside the active material differentiates the OECTs from other devices based on the field effect. Ions are transported into and out of the channel in response to changes in the applied gate potential, which modulates output drain current *I*_D_. The density of ions injected into the active material can be described by a gate capacitance *C*, which was shown by Rivnay et al.^3^ to scale with the total volume of the transistor channel. Overall, the following dependency of the transconductance of the devices in the saturation regime is found:
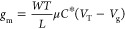
1where *W* is the channel width, *T* is the channel thickness, *L* is the channel length, μ is the hole mobility in the channel, C* is the volumetric capacitance, *V*_T_ is the threshold voltage, and *V*_g_ is the gate voltage.

Often, the *WT*/*L* ratio in eq 1 is adjusted to optimize the performance of the OECTs. However, while increasing the width and thickness of the channel is straightforward when fabricating an OECT, doing so leads to slower response times.^4^ It has been shown that ionic time constant τ is limited by the charging of gate capacitance *C* of the device and the resistance of the electrolyte, *R*_ion_, leading to a geometric dependence of τ ∝ *TWL*,^4^ resulting in a *g*_m_/τ ratio that scales as
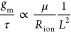
2

This dependency adds complexity to the optimization of an OECT. To design fast, high-performance, high-amplification OECTs, the distance between the drain and source electrodes (*L*) has to be minimized. However, for conventional OECTs, this is often restricted by the resolution of the lithography process, and a compromise is often made by increasing channel width *W* to obtain high transconductance while keeping the response times as fast as required.

Vertical organic electrochemical transistors (vOECTs) have garnered a considerable amount of interest as they can overcome the limitations caused by the lithography process. In vOECTs, the drain and source electrodes are placed on top of each other instead of side by side, thereby enhancing the performance while minimizing the spatial footprint. The separating distance between the electrodes is defined by either an insulation layer^5,6^ or the active material itself.^7^ The separating layer is usually deposited through a spin coating method, allowing for submicrometer channel lengths and, according to eq 2, higher transconductance and reduced switching times. However, the reduction of intrinsic channel resistance enhances the influence of parasitic resistances in series with the transistor channel, altering the expected steady state characteristics in several ways.^8^

Precisely identifying the source of these parasitic resistances is essential to reaching the intrinsic performance limit of vertical OECTs. Kaphle et al.^9^ found that OECTs can be limited by contact resistance at the metallic source/drain electrodes. Using the transmission line method, they showed that the contact resistance depends exponentially on the gate potential, which can explain the characteristic bell-shaped transconductance curve. Donahue et al.^6^ attributed the observed plateauing of max transconductance with increasing *WT*/*L* ratios, contradicting eq 1, to ohmic resistance coming from the metallic interconnect lines in series between the power supply and the semiconductor channel. Furthermore, they concluded that the overlap distance of the active material on top of the electrodes that does not participate in the electronic conduction of the device is detrimental to the on and off switching times of the device. Although documentation of parasitic resistance is found in these reports, focused research to identify the exact source has not yet been conducted.

Recently, a novel vOECT design that aims to achieve high transconductance with minimized response times was proposed. The device takes advantage of the precise control of film thickness when channels of various compositions are grown by electrodeposition.^10,11^ Previously, we were able to show that devices with PEDOT:PSS [poly(3,4-ethylenedioxythiophene)-poly(styrolsulfonate)] channels present high performance, that is, a high *g*_m_/τ ratio. However, the transconductance saturates at high geometrical *W*/*L* ratios, leading to a reduction in the *g*_m_/τ ratio.^4^

Here, we study the origin of this worsening performance at large *W*/*L* ratios. The vOECTs are characterized using a four-point measurement to minimize the influence of line resistances in series with the channel, allowing for enhanced operation of the active material. Under these enhanced conditions, electropolymerized organic films deposited under different galvanostatic conditions, under different deposition times, and using different precursor solutions are characterized. Overall, we show that the four-point measurement increases the device performance drastically. Furthermore, variances of film morphology resulting from different deposition preconditions only weakly influence the performance. It is argued that this insensitivity to the deposition conditions results in small device-to-device variations and high reproducibility of vOECTs.

## 2 Results

### 2.1 Structure of Vertical OECTs

**Figure 1:**
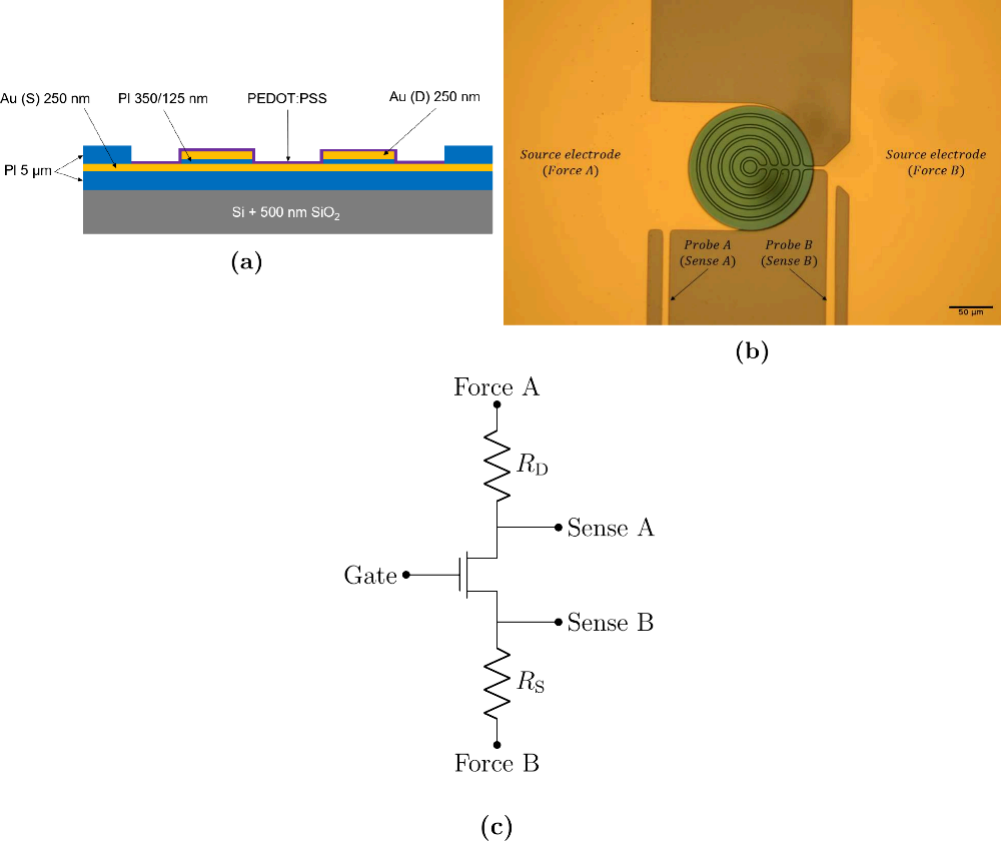
Device setup. (a) Cross section of the vertical OECT. A circular gold electrode on the bottom and a ring-shaped one on the top form the source and drain electrodes, respectively. The source and drain are separated by a 350 or 125 nm thin polyimide (PI) passivation layer, defining channel length *L*. PEDOT:PSS is grown by electropolymerization on top of the source and drain electrode. Adapted with permission from ref (4). (b) Top view of the vOECT design. To reduce the device footprint, the top electrode has a ring-shaped form. The number of rings determines channel width *W*. Each electrode has an additional thin probe structured on the same metallic layer connected to a different interconnection pad to measure the potential drop at the channel. (c) Circuit diagram of the resulting architecture.

The OECT design is described in detail in ref (4). The cross-section and top view images of the vOECTs are shown in panels a and b, respectively, of Figure 1. A cross-sectional scanning electron microscopy image is shown in Figure S1, and more morphological characterization is shown in ref (4). Ring-shaped electrodes are used to maximize channel width *W* by increasing the number of concentric rings while keeping a small footprint. A polyimide insulation layer, 350 or 125 nm thick, separates the drain and source electrodes and defines channel length *L*.

To realize the four-point structure, two additional thin probes (probes A and B in Figure 1b) are added close to the channel of the device to measure the potential drop close to the active device. As the potential is measured with a voltage probe with high input resistance, the potential applied between the source and drain can be measured without the influence of parasitic resistance coming from the metallic lines and voltage source (cf. Figure 1c). The drain-source voltage is applied through two large probes (labeled forces A and B). The sourcing strategy takes the measured potential drop at the channel and uses it as feedback by comparing it to the programmed drain voltage at the voltage source. This virtually eliminates the gate voltage-dependent resistance in series with the PEDOT:PSS channel and any other resistance influence from the source, interconnections, or wires.

The PEDOT:PSS films used in the vOECTs are electropolymerized on the surface of both source and drain electrodes simultaneously, following the methods of Cui and Martin^12^ and Starbird et al.^13^ A visible–near-infrared spectrum comparing the traditional PEDOT:PSS mixture deposited by spin coating [with additives ethylene glycol, dodecylbenzenesulfonic acid and (3-glycidyloxypropyl)trimethoxysilan]^14−16^ and the electrodeposited version used in this work is available in Figure S2. Although the electrodeposited PEDOT:PSS shows higher absorbance for wavelengths above 900 nm, indicating a polaron and bipolaron density higher than that of the spin-coated counterpart, the overall characteristic is identical.^17^

**Figure 2:**
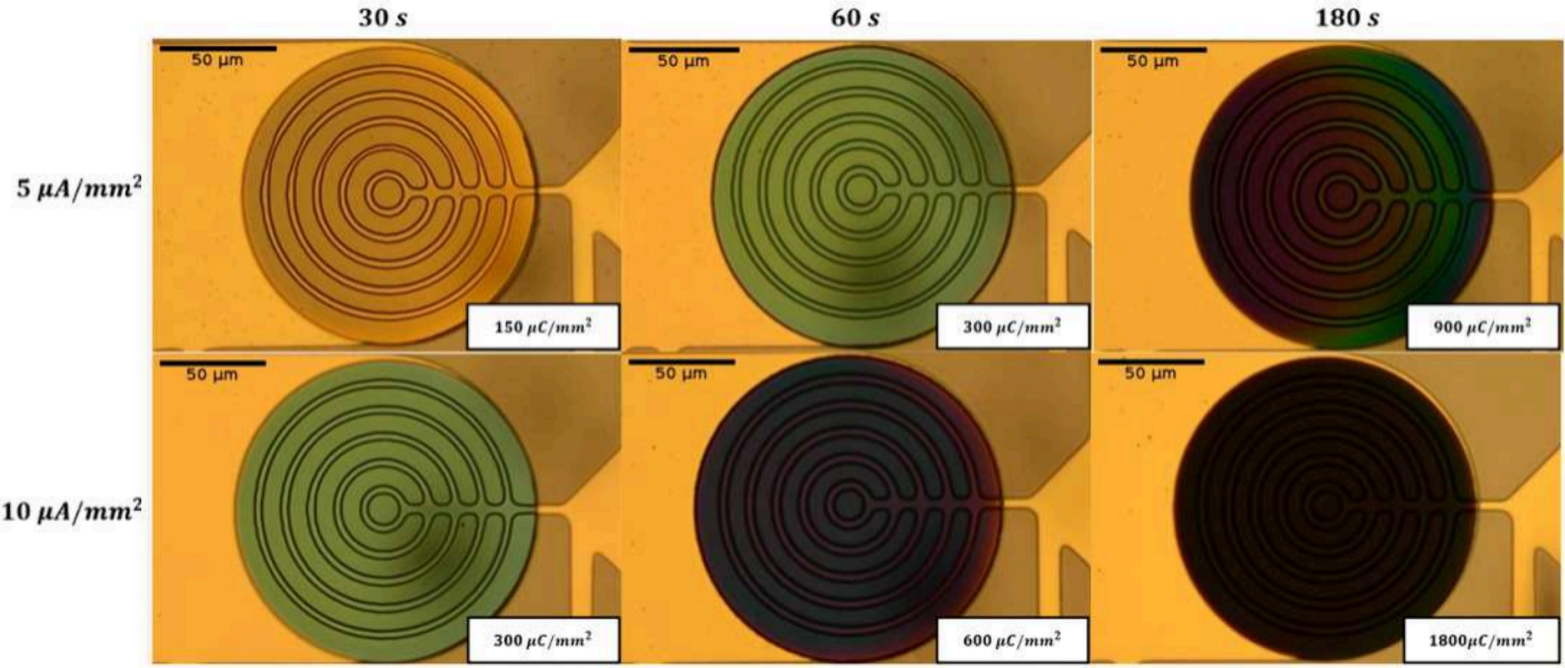
Varying the PEDOT:PSS morphology. Images of vOECTs with a channel width *W* of 1.74 mm for different electropolymerization conditions. The product of the current density and time is proportional to the thickness of the films (*T*).

The thickness of the films is varied by using different conditions during the electrodeposition of the films. The varied parameters are current density *J*, deposition time *t*, and the composition of the precursor solution. Increasing either current density *J* or deposition time *t* results in thicker channels, which is confirmed by the increase in opacity observed in Figure 2.

### 2.2 Steady State Characterization

**Figure 3:**
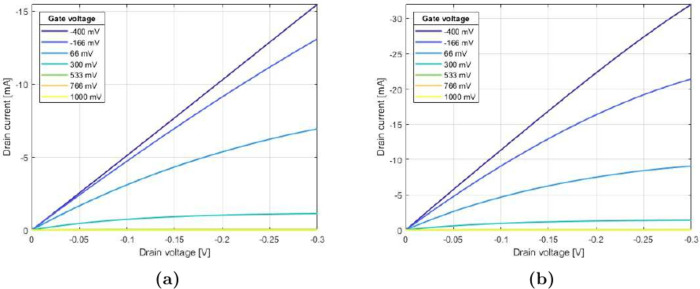
Output characteristics of vOECTs. Dependence of the drain current on the drain voltage for different constant gate voltages for a transistor with a width *W* of 660 μm and a length *L* of 350 nm that was electropolymerized for 60 s with a constant current density of 5 μA/mm^2^ measured with (a) two and (b) four points.

Figure 3 shows the output characteristics of a transistor grown by electropolymerization for 60 s at a current density *J* of 5 μA/mm^2^ with a channel width *W* of 660 μm. The measurement with two-point voltage sourcing (Figure 3a) shows that the saturation region is reached for only higher gate voltages, i.e., stronger depletion of the channel. For lower gate voltages, the drain current continues to increase linearly even at higher drain potentials, i.e.,  for most voltages (*V*_DS_, drain-source voltage; *I*_D_, drain current).

Implementing four-point sourcing (Figure 3b) not only increases the overall drain current but also improves the saturation behavior of devices. Overall, smaller drain voltages are needed to reach a behavior similar to that for the two-point measurement.

**Figure 4:**
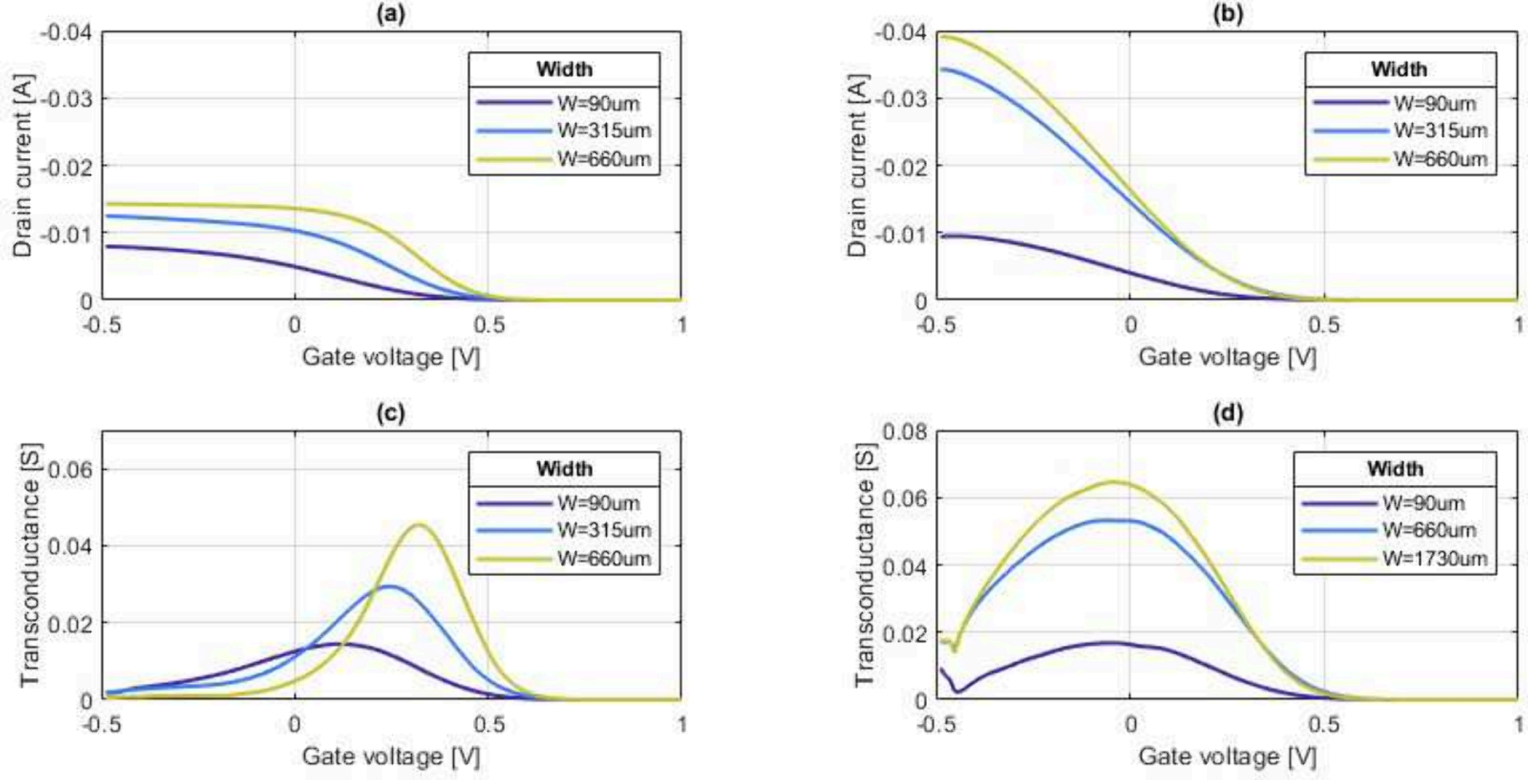
Steady state characteristics of vOECTs. Transfer characteristics for three transistors with increasing channel widths measured at a drain voltage *V*_DS_ of −0.3 V that were electropolymerized for 30 s with a constant current density of 5 μm/mm^2^, measured in the (a) two-point and (b) four-point geometries. The transistors measured with four points show an enhanced ON current. Transconductance characteristics with gate voltage measured with (c) two and (d) four points. The absolute maximum of the characteristic bell curve shifts toward higher values when measuring with two points, whereas no shift is observed in the four-point measurement geometry.

The transfer characteristics and transconductance of the vOECTs for two- and four-point measurements are shown in Figure 4. When the voltage is sourced in a standard fashion (i.e., using two probes only), drain current *I*_D_ saturates at negative gate voltages *V*_GS_. The saturation sets in earlier (that is, at less negative voltages) for wider transistor channels, leading to a shift of the characteristic toward more positive voltages for wider channels (Figure 4a).

**Figure 5:**
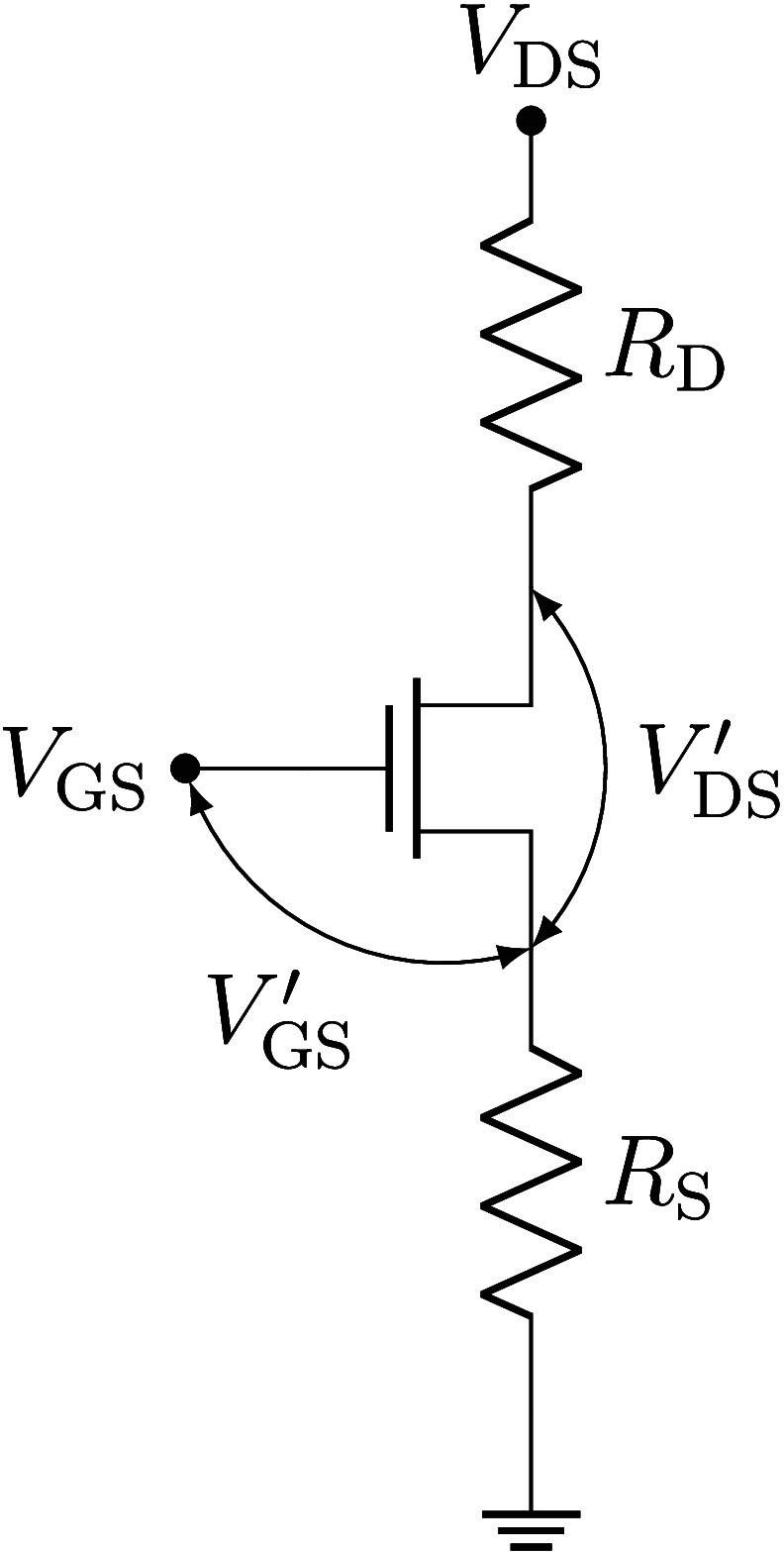
Modeling parasitic effects on device performance. Equivalent circuit used to discuss the influence of parasitic resistances *R*_D_ and *R*_S_ following the results of Chou and Antoniadis.^18^

This characteristic saturation behavior can be explained by the model of Chou and Antoniadis.^18^ The differential of the drain current (d*I*_D_) can be written as
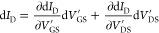
3
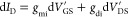
4where *V*_DS_^′^ and *V*_GS_^′^ are the internal drain-source and the gate-source voltages, respectively, that are applied to the transistor, i.e., without any voltage drop across parasitic drain *R*_D_ and source *R*_S_ resistances (cf. Figure 5).  is the intrinsic transconductance of the transistors, which is not degraded by parasitic resistances,  the drain-source conductance, which can be approximated by the slope of the output characteristics shown in Figure 3. Internal voltages *V*_DS_^′^ and *V*_GS_^′^ are related to externally applied voltages *V*_DS_ and *V*_GS_, respectively, by
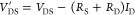
5
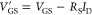
6It follows for the externally measured transconductance 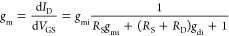
7

Equation 7 shows that ideally, the transfer characteristic is measured in the saturation regime, i.e., at *g*_di_ = 0. For diminishing parasitic resistances (*R*_S_ = *R*_D_ = 0), the measured transconductance will equal the intrinsic one, i.e., *g*_m_ = *g*_mi_.

Here, however, neither is *g*_di_ ≈ 0 nor can the series resistances at the source and drain (*R*_S_ and *R*_D_, respectively) be neglected. Whenever the drain current *I*_D_ is increased by a decrease in gate voltage *V*_GS_ (or application of a more negative *V*_GS_), internal drain voltage *V*_DS_^′^ decreases (cf. eq 5), ultimately below the pinch-off voltage. Hence, the transistor is not operating in the saturation regime anymore, and drain-source conductance *g*_di_ increases with a decrease in *V*_GS_. Once drain-source conductance *g*_di_ becomes large compared to 1/(*R*_S_ + *R*_D_), the transfer characteristic saturates and *g*_m_ decreases to zero, as indeed observed experimentally in panels a and c of Figure 4.

As one can see in Figure 4a, the saturation in the transfer characteristic sets in later for a smaller channel width. This, again, is in line with eq 7, as intrinsic transconductance *g*_mi_, channel transconductance *g*_di_, and the pinch-off voltage separating the saturation from the linear regime are expected to increase with channel width. Therefore, the transconductance levels off earlier (at less negative voltages). This effect also causes the characteristic transconductance bell curve to become narrower and shift toward the right (Figure 4c).

In contrast, when the drain-source voltage is sourced using the four-point structure, *R*_S_ and *R*_D_ can be approximately neglected. Indeed, it is observed that instead of a shift in the gate voltage of the transfer characteristic, the drain current is merely modulated by the channel width; i.e., the drain current increases with the channel width (Figure 4b). Consequently, the position of the maximum transconductance remains at the same gate voltage value, approximately −0.05 V (Figure 4d), and does not shift.

**Figure 6:**
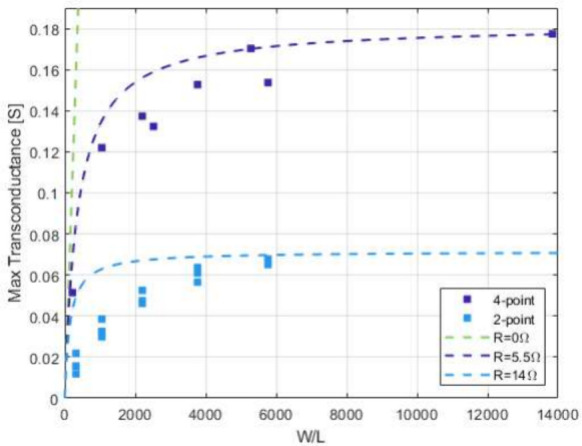
Scaling of transconductance with the *W*/*L* ratio. Dependence of the maximum transconductance on the *W*/*L* ratio for transistors electropolymerized with a current density *J* of 0.5 μA/mm^2^ for a *t* of 30 s and measured at a drain voltage *V*_D_ of −0.5 V. Symbols show *g*_m,max_ values for four- and two-point sourcing. The dashed lines are simulations of an OECT according to the Bernards model^19^ with a hole mobility μ of 2 cm^2^ V^–1^ s^–1^, a volumetric capacitance C* of 50 F cm^–3^, a threshold voltage *V*_T_ of 0.5 V, and a thickness *T* of 100 nm, including the effect of parasitic resistance (cf. eq 8). It is assumed that the maximum transconductance is found at a *V*_GS_^′^ of −0.05 V (cf. Figure 4d). The maximum transconductance data measured with four points can be fitted by the model data more accurately than the data measured with two points.

Peak transconductance *g*_m,max_ is plotted versus geometric ratio *W*/*L* in Figure 6. The peak transconductance shows an almost linear increase at low *W*/*L* ratios but levels off for larger *W*/*L* ratios. The apparent increase in *g*_m,max_ with *W*/*L* for *W*/*L* values of <1000 is larger for the four-point measurement, whereas the saturation of *g*_m,max_ happens earlier, i.e., at smaller *W*/*L* ratios.

The data shown in Figure 6 can be modeled according to eq 7. As the peak transconductance is plotted, i.e., before the transconductance decreases because of the drain-source conductance, the approximation *g*_di_ ≪ 1/(*R*_S_ + *R*_D_) is used.^18^
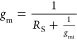
8

The data measured with four points fit the model with great accuracy; i.e., the assumption of a small drain-source conductance is justified. The remaining parasitic source resistance amounts to 5.5 Ω, and a channel thickness *T* of 100 nm is assumed. However, the approximation does not work as well for the two-point measurements. Here, the remaining *g*_di_ (cf. Figure 3) is still significant at low *W*/*L* ratios, leading to a stronger influence of parasitic resistance.

Figure 6 shows that only by using the four-point measurement setup can the intrinsic performance of vOECTs be reached, at least at small *W*/*L* ratios. Hence, in the following, we try to optimize this performance by optimizing the electropolymerization conditions.

**Figure 7:**
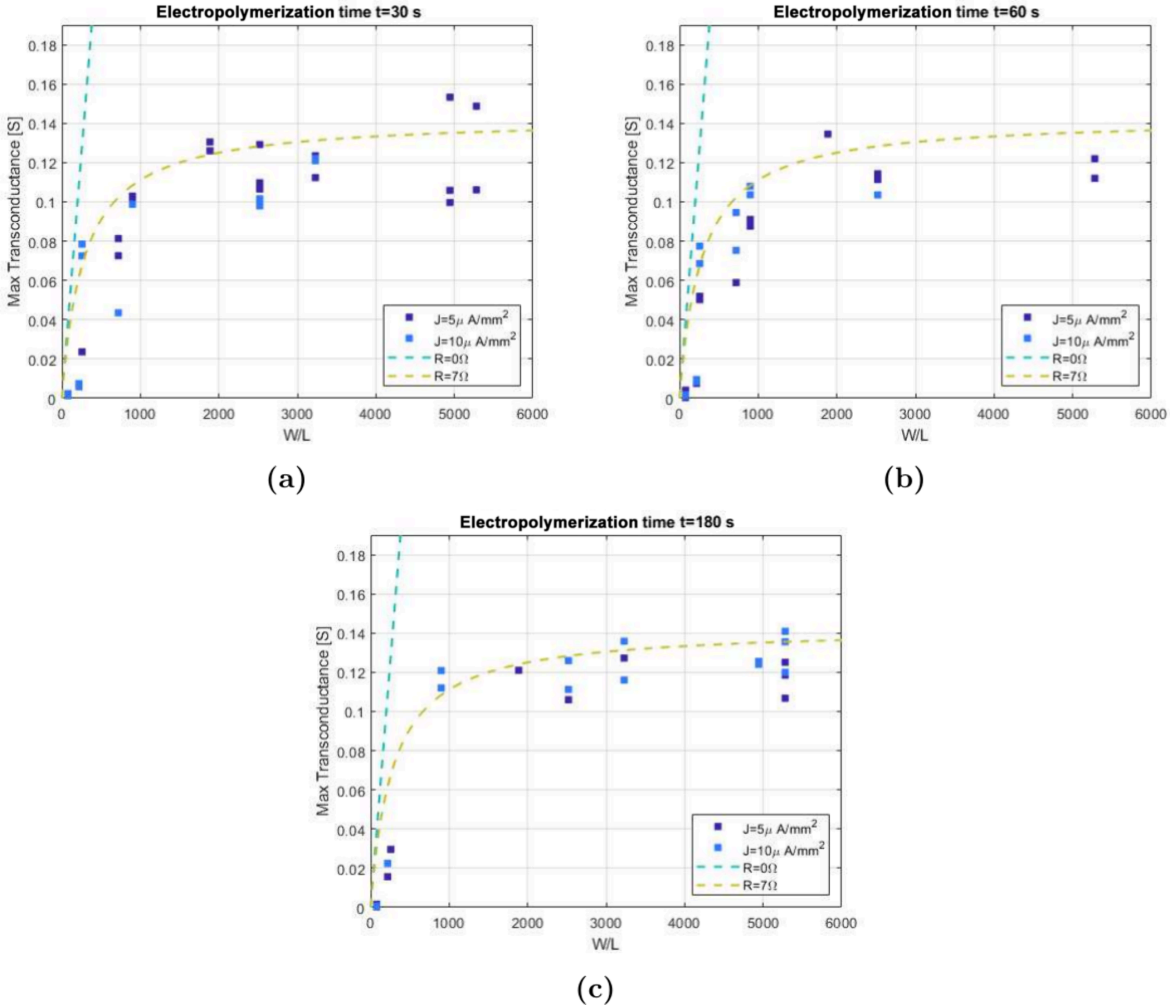
Scaling of transconductance with *W*/*L* ratio under different electropolymerization conditions. Dependency of maximum transconductance on current density and *W*/*L* ratio for transistors electropolymerized for (a) 30, (b) 60, and (c) 180 s. The data are obtained at a drain voltage *V*_D_ of −0.5 V. The lines plotted are simulations of an OECT according to the Bernards model^19^ with a hole mobility μ of 2 cm^2^ V^–1^ s^–1^, a volumetric capacitance C* of 50 F cm^–3^, a threshold voltage *V*_T_ of 0.5 V, and a thickness *T* of 100 nm. The considered gate potential (*V*_GS_^′^) is −0.05 V.

Figure 7 shows the dependency of transconductance on the *W*/*L* ratio for transistors electropolymerized with different deposition current densities and times. All of these measurements were taken using four-point voltage sourcing. For all variations, a plateau at *W*/*L* ratios of >1000 at a *g*_m,max_ of ∼140 mS is found. The data can as well be fitted by eq 8 using a source resistance *R*_S_ of 7 Ω and a thickness *T* of 100 nm. While different deposition parameters are used, all data sets follow a similar trend. This suggests that for this architecture, a deposition time of 30 s is sufficient to produce a channel with the maximum amplification capabilities. Furthermore, there is no clear difference between films deposited by using different current densities *J*. Overall, this result suggests that using longer deposition durations and higher current densities will result in thicker films (Figure 2) that, however, do not show any increase in transconductance. This is in line with our previous report,^4^ which showed that the current transport in these vOECTs is limited to a small cavity between the source and drain electrode. Once this cavity is filled, a maximum transconductance is reached.

## 3 Four-Point Measurement to Benchmark the Performance of Different Geometries

Vertical organic electrochemical transistors are attracting attention for being a straightforward approach for improving the *g*_m_/τ ratio, as it is expected to inversely scale with the square of the channel length. The four-point sourcing approach to characterize vOECTs as shown here ensures that results from different OECT designs can be compared fairly. Using the *g*_m_/τ ratio as a figure of merit, the best performing transistors are obtained here for a channel width *W* of 90 μm (i.e., for devices with only one drain ring), reaching a level of  (*g*_m_ = 79 mS, τ_on_ = 275 μs, and τ_off_ = 94 μs). This level is almost twice that previously reported.^4^ Increasing the channel width by the addition of more rings (cf. Figure 1b) increases *g*_m,max_, but the gap between each ring adds to the channel capacitance and possibly adds parasitic resistance leading to a decrease in *g*_m_/τ.

Vertical architectures are good for not only strong amplification capabilities and fast response times but also reducing the footprint of devices. For example, Facchetti et al.^7^ normalized the transconductance of their vOECTs with the footprint area, reaching a *g*_m,A_ of 226 μS μm^–2^. When our results are normalized to their footprint, the best vOECTs reach a *g*_m,A_ of 51 μS μm^–2^. Because this architecture takes advantage of the cavity created between the drain and source electrodes during the lithography process, the footprint can be optimized by simplifying the device design, which is expected to result in an improvement in both mentioned figures of merit (*g*_m_/τ and *g*_m,A_).

## 4 Conclusion

Exploring the limits of channel length scaling in vertical OECTs comes with the stronger influence of parasitic resistance. Understanding their effects on performance is important, as it allows for a better definition of design rules for OECTs. Here, we have successfully presented an experiment that can virtually eliminate most of the parasitic line resistance, allowing the measurements to be affected by only a small series resistance, which might stem from the contact resistance of the active material or remaining resistances of the metallic electrodes.

Using the four-point measuring technique, we can for the first time characterize the intrinsic performance of the vOECT, which in two-point measurements is always degraded by parasitic effects. This allows us to study the influence of deposition conditions (or any differences in the organic semiconductor) on device performance, in particular, the deposition current density and deposition times. Although these parameters are important for controlling the film thickness, they do not affect the transconductance of our devices. This is in line with previous results^4^ that show that only a small cavity between the source and drain electrode is participating in the behavior of the drain current.

Overall, this approach allows for closer observation of the intrinsic behavior of the active material in the short channels of vOECTs, free of external factors. We expect that the result can be extended to other material systems, e.g., spin-coated materials. However, the limitation from parasitic resistances might become relevant at larger potentials only, in particular for accumulation type OECTs, which tend to show a smaller transconductance *g*_m_ and better saturation behavior (i.e., higher *g*_di_). Therefore, more engineering studies on the improvement of contact conductivity on OECTs need to be performed to further overcome the limiting factors on their performance, as has been done for organic field-effect transistors (OFETs).^20−23^

## 5 Methods

### 5.1 Device Fabrication

The vOECTs are structured on 4 in. silicon wafers coated with a 500 nm thick silicon oxide layer in the IMSAS ISO-6 cleanroom. First, a 5 μm thick polyimide layer (U-Varnish S, UBE Corp.) is spin-coated and cured using a vacuum hot plate to create a base insulation layer. A 250 nm thick gold layer is afterward deposited and structured using magnetron dc sputtering (Pro Line PVD 75, Kurt J. Lesker Company Ltd.), photolithography (AZ 1518, MicroChemicals GmbH), and wet chemical etching (Au etch 200, NB Technologies GmbH) to create the source electrodes and sense A probes. Then, two thin layers of polyimide of 350 or 125 nm are spin-coated and cured to insulate the source electrodes and define the channel length of the transistor. A second gold layer is deposited using the same processes described above to structure the drain electrodes and sense B probes. To insulate the conducting paths and define the area for electropolymerization of PEDOT:PSS, another 5 μm thick polyimide layer is spin-coated and cured. A short (30 s) oxygen RIE plasma treatment (STS ICP) is performed directly before coating of polyimide to significantly improve the adhesion between consecutive polyimide layers. The top insulation layer is then structured together with the thin polyimide layer by using photolithography (AZ 10XT, MicroChemicals GmbH) and oxygen RIE plasma (STS ICP). After wafer dicing, the electropolymerization process is performed. For electrodeposition of PEDOT:PSS, a monomer solution of 10 mM EDOT (3,4-ethylenedioxythiophene, Sigma-Aldrich) and 2 wt % NaPSS [poly(sodium 4-styrenesulfonate), MW 70 000, Sigma-Aldrich] dissolved in deionized (DI) water is used. Galvanostatic conditions of 5 or 10 μA/mm^2^ are applied between the samples and the platinum counter electrode. For a more homogeneous coating, the monomer solution is stirred slowly using a magnetic stirrer. The source and drain electrodes are coated simultaneously with PEDOT:PSS. After coating, the samples are cleaned in DI water and dried with nitrogen. The coating thickness is controlled by the electropolymerization time and current density.

### 5.2 Electrical Characterization

Four-point probe measurements were performed with a Keithley Series 2400 SourceMeter, configured with the four-wire remote sensing setup in one SMU to source the drain voltage. A voltage sweep was configured and supplied by the second SMU to an ∼1 cm^2^ PEDOT:PSS gold gate electrode to obtain the transfer characteristics using Ringer’s solution (B. Braun) as the electrolyte. The transconductance was determined by a central difference derivative and a moving average to reduce the noise.

Transient measurements were performed with a Tektronix oscilloscope using an amplifier circuit to convert and amplify measured currents to voltage signals. On and off switching times were determined by the time to switch from the ON drain current to 63% of the OFF current and vice versa.

## Supporting Information Available

Additional characterization of materials and devices, including a cross section of the vOECT channel and visible–near-infrared spectra of electropolymerized PEDOT:PSS (PDF)

## References

[ref1] Sophocleous M.; Contat-Rodrigo L.; García-Breijo E.; Georgiou J. Organic Electrochemical Transistors as an Emerging Platform for Bio-Sensing Applications: A Review. IEEE Sensors Journal 2021, 21, 3977–4006. 10.1109/JSEN.2020.3033283.

[ref2] Tan S.; Keene S.; Giovannitti A.; Melianas A.; Moser M.; McCulloch I.; Salleo A. Operation Mechanism of Organic Electrochemical Transistors as Redox Chemical Transducers. Journal of Materials Chemistry C 2021, 9, 12148–12158. 10.1039/D1TC02224E.

[ref3] Rivnay J.; Leleux P.; Ferro M.; Sessolo M.; Williamson A.; Koutsouras D. A.; Khodagholy D.; Ramuz M.; Strakosas X.; Owens R. M.; Benar C.; Badier J.-M.; Bernard C.; Malliaras G. G. High-performance transistors for bioelectronics through tuning of channel thickness. Sci. Adv. 2015, 1, e1400251 10.1126/sciadv.1400251. 26601178 PMC4640642

[ref4] Skowrons M.; Schander A.; Negron A. G. P.; Lüssem B. The Trade-Off between Transconductance and Speed for Vertical Organic Electrochemical Transistors. Adv. Electron. Mater. 2024, 10, 2300673 10.1002/aelm.202300673.

[ref5] Brodský J.; Gablech I.; Migliaccio L.; Havlíček M.; Donahue M. J.; Głowacki E. D. Downsizing the Channel Length of Vertical Organic Electrochemical Transistors. ACS Appl. Mater. Interfaces 2023, 15, 27002–27009. 10.1021/acsami.3c02049. 37216209 PMC10251347

[ref6] Donahue M. J.; Williamson A.; Strakosas X.; Friedlein J. T.; McLeod R. R.; Gleskova H.; Malliaras G. G. High-Performance Vertical Organic Electrochemical Transistors. Adv. Mater. 2018, 30, 1705031 10.1002/adma.201705031. 29266473

[ref7] Huang W.; Chen J.; Yao Y.; Zheng D.; Ji X.; Feng L.-W.; Moore D.; Glavin N.; Xie M.; Chen Y.; Pankow R.; Surendran A.; Wang Z.; Xia Y.; Bai L.; Rivnay J.; Ping J.; Guo X.; Cheng Y.; Marks T. J.; Facchetti A. Vertical organic electrochemical transistors for complementary circuits. Nature 2023, 613, 496–502. 10.1038/s41586-022-05592-2. 36653571 PMC9849123

[ref8] Friedlein J. T.; McLeod R. R.; Rivnay J. Device physics of organic electrochemical transistors. Org. Electron. 2018, 63, 398–414. 10.1016/j.orgel.2018.09.010.

[ref9] Kaphle V.; Liu S.; Al-Shadeedi A.; Keum C.-M.; Lüssem B. Contact Resistance Effects in Highly Doped Organic Electrochemical Transistors. Adv. Mater. 2016, 28, 8766–8770. 10.1002/adma.201602125. 27511804

[ref10] Koutsouras D. A.; Torricelli F.; Blom P. W. Submicron Vertical Channel Organic Electrochemical Transistors with Ultrahigh Transconductance. Adv. Electron. Mater. 2023, 9, 2200868 10.1002/aelm.202200868.

[ref11] Lee J.; Chhatre S.; Sitarik P.; Wu Y.; Baugh Q.; Martin D. C. Electrochemical Fabrication and Characterization of Organic Electrochemical Transistors Using poly(3,4-ethylenedioxythiophene) with Various Counterions. ACS Appl. Mater. Interfaces 2022, 14, 42289–42297. 10.1021/acsami.2c10149. 36095248

[ref12] Cui X.; Martin D. C. Electrochemical deposition and characterization of poly(3,4-ethylenedioxythiophene) on neural microelectrode arrays. Sens. Actuators, B 2003, 89, 92–102. 10.1016/S0925-4005(02)00448-3.

[ref13] Starbird R.; Bauhofer W.; Meza-Cuevas M.; Krautschneider W. H. Effect of experimental factors on the properties of PEDOT-NaPSS galvanostatically deposited from an aqueous micellar media for invasive electrodes. The 5th 2012 Biomedical Engineering International Conference, 2012; pp 1–5 10.1109/BMEiCon.2012.6465500.

[ref14] Paudel P. R.; Dahal D.; Radha Krishnan R. K.; Skowrons M.; Lüssem B. Top-contact organic electrochemical transistors. AIP Adv. 2022, 12, 045310 10.1063/5.0087638.

[ref15] Colucci R.; Feitosa B. d. A.; Faria G. C. Impact of Ionic Species on the Performance of Pedot:PSS-Based Organic Electrochemical Transistors. Adv. Electron. Mater. 2024, 10, 2300235 10.1002/aelm.202300235.

[ref16] Matrone G. M.; Bruno U.; Forró C.; Lubrano C.; Cinti S.; van de Burgt Y.; Santoro F. Electrical and Optical Modulation of a PEDOT:PSS-Based Electrochemical Transistor for Multiple Neurotransmitter-Mediated Artificial Synapses. Adv. Mater. Technol. 2023, 8, 2201911 10.1002/admt.202201911.

[ref17] Barbosa H. F. P.; Higuita G. D. G.; Günther F.; Faria G. C. Tunable Charge-Density PEDOT:PSS for Application in Post-Synaptic Organic Neuromorphic Electrodes. Adv. Electron. Mater. 2022, 8, 2100864 10.1002/aelm.202270005.

[ref18] Chou S.; Antoniadis D. Relationship between measured and intrinsic transconductances of FET’s. IEEE Trans. Electron Devices 1987, 34, 448–450. 10.1109/T-ED.1987.22942.

[ref19] Bernards D.; Malliaras G. Steady-State and Transient Behavior of Organic Electrochemical Transistors. Adv. Funct. Mater. 2007, 17, 3538–3544. 10.1002/adfm.200601239.

[ref20] Vanoni C.; Tsujino S.; Jung T. A. Reduction of the contact resistance by doping in pentacene few monolayers thin film transistors and self-assembled nanocrystals. Appl. Phys. Lett. 2007, 90, 193119 10.1063/1.2738382.

[ref21] Liu C.; Xu Y.; Noh Y.-Y. Contact engineering in organic field-effect transistors. Mater. Today 2015, 18, 79–96. 10.1016/j.mattod.2014.08.037.

[ref22] Singh S.; Mohapatra S. K.; Sharma A.; Fuentes-Hernandez C.; Barlow S.; Marder S. R.; Kippelen B. Reduction of contact resistance by selective contact doping in fullerene n-channel organic field-effect transistors. Appl. Phys. Lett. 2013, 102, 153303 10.1063/1.4802237.

[ref23] Günther A. A.; Sawatzki M.; Formánek P.; Kasemann D.; Leo K. Contact Doping for Vertical Organic Field-Effect Transistors. Adv. Funct. Mater. 2016, 26, 768–775. 10.1002/adfm.201504377.

