# Supplementary material for: Approaching the Intrinsic Limits of Short Channel Vertical Organic Electrochemical Transistors
Source: ACS Appl Mater Interfaces. 2024 Aug 20;16(34):45234–41. doi: 10.1021/acsami.4c02772 (PMC11367573; doi:10.1021/acsami.4c02772)
Supplement: Supplementary file 1 — am4c02772_si_001.pdf [file am4c02772_si_001.pdf]

Supporting Information: Approaching the intrinsic limits of short channel vertical Organic Electrochemical Transistors

Alvaro Galeana Perez Negron,*,† Andreas Schander,† Michael Skowrons,‡ Henrique Frulani de Paula Barbosa,† and Björn Lüssem*,†

†*Institut für Mikrosensoren, -Aktoren, und -Systeme (IMSAS), Universität Bremen, 28359 Bremen, Germany*

‡*Department of Physics, Kent State University, Kent, OH, 44240*

E-mail: agaleana@uni-bremen.de; bluessem@imsas.uni-bremen.de

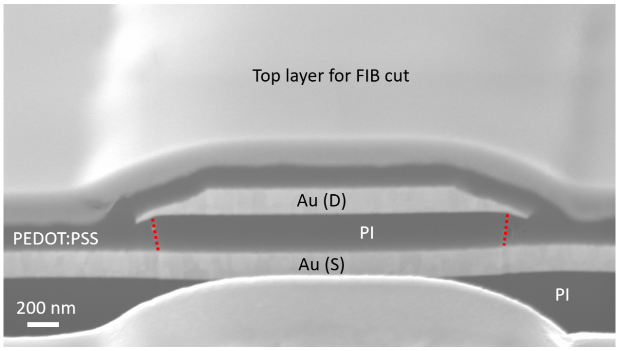

Figure S1: **Crossection of vOECT channel:** Crossection of the vOECT channel after electrodepositon of PEDOT:PSS (120s). The source and drain electrodes are separated by a polyimide passivation layer.

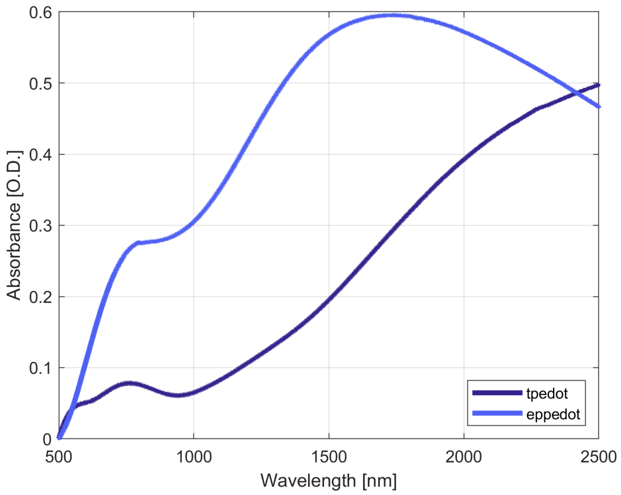

Figure S2: **Vis-NIR spectra:** Vis-NIR spectra of spin-coated PEDOT:PSS (tpedot) and electropolymerized PEDOT:PSS (eppedot) used in this work. eppedot seems to present higher charge densities (polarons and bipolarons), given the higher absorbance for wavelengths above 900 nm.
